# Effects of early medication treatment and metformin use for cancer prevention in diabetes patients: a nationwide sample cohort study in Korea using extended landmark time analysis

**DOI:** 10.4178/epih.e2021103

**Published:** 2021-12-17

**Authors:** Hwa Jeong Seo, Hyun Sook Oh

**Affiliations:** 1 Medical Informatics and health Technology (MIT), Department of Health Care Management, Gachon University, Seongnam, Korea; 2 Department of Applied Statistics, School of Social Science, Gachon University, Seongnam, Korea

**Keywords:** Type 2 diabetes mellitus, Anticancer agents, Medication compliance, Metformin, Drug prescription

## Abstract

**OBJECTIVES:**

This study investigated the effectiveness of early medication treatment and metformin use for cancer prevention in type 2 diabetes patients.

**METHODS:**

Population-based cohort data were used from the Korean National Health Insurance Service-National Sample Cohort database (KNHIS-NSC) for 2002–2013. Patient-specific medication prescription status was defined by the landmark time (LMT; a fixed time after cohort entry), considering both pre- and post-LMT prescriptions to control methodological biases in observational research. The LMT was set to 2 years. Logistic regression analysis with multivariable adjustment was conducted to analyze cancer incidence by patient-specific medication prescription status.

**RESULTS:**

Only 33.4% of the subjects were prescribed medication early (before the LMT) with compliance. Cancer incidence in individuals with early prescription and compliance was 25% lower (odds ratio [OR], 0.75; 95% confidence interval [CI], 0.67 to 0.84) than in those without. As early-prescribed medications, metformin monotherapy and metformin combination therapy were associated with 34% (OR, 0.66; 95% CI, 0.51 to 0.83) and 25% (OR, 0.75; 95% CI, 0.64 to 0.88) lower cancer risk than non-use, respectively. Patients who were prescribed late (post-LMT) but did not comply with the prescription had a 24% (OR, 1.24; 95% CI, 0.97 to 1.58) higher cancer incidence than non-users. Among patients who started monotherapy early without changes throughout the entire follow-up period, those who started on metformin had a 37% (OR, 0.63; 95% CI, 0.41 to 0.99) lower risk of cancer than non-metformin users.

**CONCLUSIONS:**

Doctors must prescribe antidiabetic medication early, and patient compliance is required, regardless of the prescription time, to prevent cancer. Metformin monotherapy or combination therapy is recommended as an early prescription.

## INTRODUCTION

The global prevalence of diabetes is rising to epidemic proportions. It was estimated that 463 million people had diabetes in 2019, and the number was predicted to increase by 25% in 2030 and 51% in 2045 [1,2]. Diabetes patients are more likely to develop multiple types of cancer, including gastroenterological, colorectal, liver, breast and pancreatic cancer, than individuals without diabetes [3–5]. Cancer is one of the most important causes of death; the incidence of cancer is increasing worldwide and is expected to increase in the future, making it a significant component of the worldwide burden of disease [6].

Immediate treatment (lifestyle modification and medication) for newly diagnosed patients may be necessary to avoid diabetes complications including cancer [7–9]. It is well known that lifestyle changes such as exercise, diet, and walking are very important after the diagnosis of diabetes. However, in an analysis of large population-based sample data, only minimal changes were reported in the lifestyles of patients with diabetes [10]. Therefore, pharmacological treatment is important for diabetes management. However, poor compliance with prescribed medication is a significant problem in healthcare [11,12]. It was reported that compliance with chronic medications was often lower than compliance with acute medications [11,13].

Metformin, a drug used to treat type 2 diabetes, is the most widely prescribed diabetes medication worldwide because of its outstanding insulin regulatory effect, low cost, suppression of diabetes complications, and anticancer effects [14–16]. An observational study in 2005 demonstrated that metformin had an inhibitory effect on all cancer types (23% reduction in the incidence of any cancer) [17], and since then, research interest in the anticancer and therapeutic effects of metformin has increased. Numerous studies claim that metformin could suppress the growth of cancer cells in various cancers [18–24]. However, there is insufficient evidence based on the exact mechanisms or randomized controlled trials [25,26]. Several recent studies reported no significant association between metformin and cancer, and the topic remains controversial [27–30]. Most studies on the cancer-preventive effects of metformin have been observational studies with methodological biases, which was pointed out as a problem.

Immortal time bias, which is the most frequently observed bias in cohort studies, involves misclassifying drug-free time as exposure time [31–33]. Immortal time refers to the time from the cohort entry to the date of treatment or event [34]. Selection bias may arise since the target of investigation on treatment effects is the surviving patients during the immortal time. The landmark time (LMT) method developed by Anderson et al. [35] is a known method for controlling immortal time bias [36,37].

In this study, medication prescription status and compliance were investigated by considering both pre- and post-LMT prescription medications for each patient, and the effect of early medication treatment and metformin use on cancer prevention in type 2 diabetes patients was analyzed.

## MATERIALS AND METHODS

### Database

This study used the Korean National Health Insurance Service-National Sample Cohort database (KNHIS-NSC) for 2002–2013, which is a population-based retrospective cohort among the general population in Korea based on insurance eligibility for 12 years from 2002–2013 [38]. The data were anonymized and de-identified prior to the current analysis.

The qualification database contains patients’ demographic data such as personal registration number, sex, age, date of death, and income quartiles. The healthcare database contains detailed information on invoices, disease descriptions, and medication prescriptions. The names of the primary and secondary diseases were coded based on the Korean Standard Classification of Diseases. A disease description includes the disease name, invoice, and date of medication prescription. The details of the prescription include specific information about the prescribed drug, and a single invoice may describe several drugs. The generic code for a prescribed drug in the invoice is classified as the main formulation code, where the first 4 digits of the code indicate the main ingredient. Thus, any drugs sharing the same first 4 digits may be considered identical drugs. The health-examination database contains data on the results of physical examinations, drinking habits, and smoking status [39].

### Study sample

Sampling was carried out as follows: Patients diagnosed with the code of type 2 diabetes twice as either the primary or secondary disease in the healthcare database (105,763 patients) were considered as having a confirmed diagnosis of type 2 diabetes. The time of the first diagnosis was defined as the time of cohort entry (index date).

Patients with index dates of 2002 or 2003 were excluded (36,515 patients). This is because the data were extracted by stratifying the population of 1 million in 2004 based on their baseline conditions in the 2002 and 2003 data [38]. Furthermore, patients aged <30 years at the index date (1,862 patients) and those without health-examination data were also excluded (10,427 patients).

The development of cancer was defined as newly registered diagnoses of any malignancy (C00–C96) (primary cancer) according to the International Classification of Diseases, 10th revision diagnostic system [27]. The analysis of cancer incidence in this study included all cancer types except non-melanoma skin cancer (55 patients) according to the international rules of cancer registries [40].

**Figure 1:**
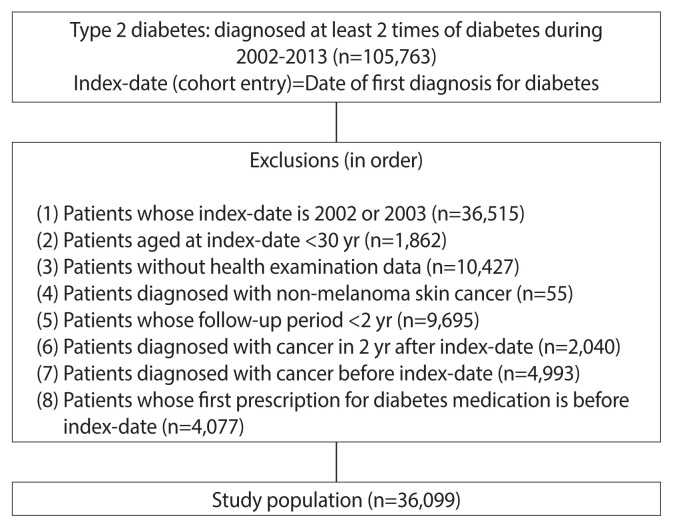
Flowchart of the sampling procedure.

Cancer takes a long period until onset and diabetes is a chronic disease with a relatively slow progression. The sample subjects were newly diagnosed patients with diabetes, so patients with a follow-up period <2 years were excluded (9,695 patients) [40]. To rule out the possibility of cancer-causing diabetes, 2,040 patients with cancer incidence within 2 years of diabetes diagnosis and 4,993 patients with cancer before the index date were excluded [40]. In addition, to investigate the medication effect in new users, 4,077 current users with the date of first diabetes medication prescription preceding the index date were excluded [34]. Thus, the final analysis targeted 36,099 subjects (Figure 1).

### Assessment of medication prescription status

The LMT was set to 2 years after cohort entry since cancer takes a long time to develop. Patients who were prescribed prior to the LMT were considered early prescription patients. If a certain drug was prescribed early and the total number of prescription days for these prescriptions was at least 180 days, it was considered sufficient drug exposure with prescription compliance, as most clinical trials on these medications were 24 weeks or longer and other studies have used a similar exposure cut-point [41,42].

Metformin monotherapy was defined as metformin monotherapy with compliance prior to LMT, and non-metformin monotherapy was defined as cases where 1 non-metformin medication with compliance prior to LMT. Non-metformin drugs included sulfonylureas, thiazolidinediones, alpha-glucosidase inhibitors (AGIs), meglitinides, and dipeptidyl peptidase 4 inhibitors, of which sulfonylureas and AGIs were the most common. Metformin combination therapy was defined as prescriptions with metformin and non-metformin drugs together or sequentially. Non-metformin combination therapy was defined as prescriptions of 2 or more non-metformin drugs simultaneously or sequentially. A list of medications with generic codes is given in Supplementary Material 1.

Patients who had pre-LMT medication prescriptions, but <180 total prescription days for each prescribed medication, were defined as having early prescription and poor compliance.

**Figure 2:**
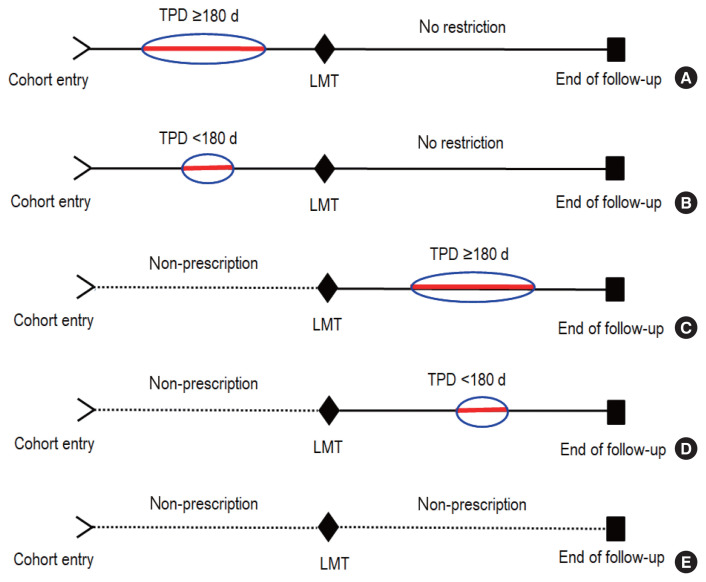
Medication prescription status; (A) early prescription with compliance, (B) early prescription with poor compliance, (C) late prescription with compliance, (D) late prescription with poor compliance, and (E) no prescription ever. TPD, total prescription days; LMT, landmark time.

Patients with no medication prescription within LMT were divided into 3 groups considering their post-LMT prescription medications and compliance: patients with no prescription after the LMT, that is, non-prescription for the entire follow-up period (no prescription ever), patients who had their first prescription after the LMT with ≥180 total prescription days (late prescription with compliance), and patients who had their first prescription after the LMT but with <180 total prescription days for each prescribed medication (late prescription with poor compliance). Figure 2 presents an illustration.

Furthermore, patients with metformin monotherapy with compliance before the LMT were classified as receiving complete metformin monotherapy if no other drugs other than metformin were prescribed for ≥180 days after the LMT; otherwise, patients were classified as receiving incomplete metformin monotherapy. Likewise, patients with non-metformin monotherapy before the LMT were classified as receiving complete non-metformin monotherapy if no other drugs other than the same drug were prescribed for ≥180 days after the LMT; otherwise, they were classified as receiving incomplete non-metformin monotherapy.

### Covariates

Age, sex, and income level were considered based on the data recorded at the time of cohort entry. Body mass index (BMI), fasting blood glucose (FBG), total cholesterol, hypertension, and serum glutamic pyruvic transaminase (alanine aminotransferase) (SGPT [ALT]) were measured as the median of the recorded values during the follow-up period. The Charlson comorbidity index (CCI) and smoking status were measured using the data from the entire follow-up period. The follow-up period from index time to completion of monitoring was considered. Waist circumference and triglycerides were not used in this study, as they have only been recorded since 2008. Drinking habits were not analyzed because there were too many missing data points. Serum glutamate oxaloacetate transaminase (aspartate aminotransferase) was not used to prevent multicollinearity since it is closely correlated with SGPT (ALT).

### Statistical analysis

The LMT method was applied to classify the prescription status of diabetes medications of each patient. The LMT method was developed by Anderson et al. [35]. A fixed time after cohort entry is chosen as the LMT, and patients are classified according to whether they received treatment or what treatment was received before the LMT. Thus, this method controls immortal time bias by limiting the immortal time within the LMT. In this study, the LMT was set to 2 years after cohort entry since cancer takes a long time to develop and diabetes is a chronic disease with relatively slow progression. Patients who had cancer before the LMT were excluded, which was reasonable because they had the potential for cancer-causing diabetes [40]. In addition, we investigated post-LMT prescription status for patients with non-prescriptions within the LMT.

The relationship of factors associated with cancer incidence was analyzed using logistic regression models, which generated odds ratios (ORs) and 95% confidence intervals (CIs). We used ORs instead of hazard ratios (HRs) since the OR is a good approximation of the cancer risk ratio because the cancer incidence is rare, whereas the HR in a Cox proportional hazards model requires proportional hazards assumption over time (all data groups must show a roughly linear relationship between cancer incidence and time) [44,45]. Most significance tests were based on 2-tailed probabilities at a significance level of 0.05, unless otherwise specified. For all statistical analyses, R version 4.2 (R Core Team, Vienna, Austria) was used.

### Ethics statement

This study was approved by the Gachon University Institutional Review Board, Seongnam, Korea (IRB File No. 2020-198).

## RESULTS

### Sample characteristics

The characteristics of the sample, which contained 36,099 individuals, are summarized in Table 1. Male accounted for 52.6% of the sample. Regarding age, 29.6% were aged 30–49 years, and 56.7% were aged 50–69 years. For BMI, 26.2% were overweight, and 47.1% were obese. 26.8% showed high FBG (>126 mg/dL), 11.7% high total cholesterol (≥240 mg/dL), and 16.0% high SGPT (ALT) (≥40 U/L). Over half of the participants (54.8%) had hypertension. The CCI score was 1 in 31.1% of participants, 2 in 12.6%, and 3 in 23.0%. The follow-up period was <5, 5–7, 7–9, and ≥9 years in 33.8%, 24.0%, 28.1% and 14.1% of the participants, respectively. Furthermore, 1,883 (5.2%) patients developed cancer.

### Medication prescription status

The diabetes medication prescription status of 36,099 subjects was examined (Table 2). The insulin group comprised 434 (1.2%) subjects. Administration of metformin monotherapy was observed in 2,536 (7.1%) patients, of which complete metformin monotherapy was found in 1,729 (68.2%). Non-metformin monotherapy was present in 3,053 (8.6%) patients, of whom 1,160 (38.0%) received complete non-metformin monotherapy. Metformin and non-metformin combination therapy was found in 5,836 (16.2%) and 473 (1.3%) patients, respectively. Early prescription and poor compliance and no prescription ever were found in 4,423 (12.2%) and 15,101 (41.8%) patients, respectively. Furthermore, 4,243 (11.8%) patients had their first prescription after the LMT, of which late prescription with compliance was found in 3,161 (74.5%) patients and late prescription with poor compliance in 1,082 (25.5%) patients.

The incidence of cancer was as follows, in ascending order: metformin monotherapy: 3.1% (complete: 2.4%, incomplete: 4.8%); metformin combination: 3.9%; no prescription ever: 5.2%, non-metformin monotherapy: 5.4% (complete: 4.9%, incomplete: 5.7%); early prescription and poor compliance: 5.4%, non-metformin combination: 5.7%, late prescription with compliance: 7.0%, insulin: 7.4%; and late prescription with poor compliance: 7.4%.

### Analysis of cancer incidence by medication prescription status

Logistic regression models were used to analyze cancer incidence according to the prescription status of drugs other than insulin as follows: first, pre-LMT prescription (with total prescription days ≥180) versus otherwise (model 1); second, all medication statuses versus no prescription ever (model 2); third, complete metformin monotherapy versus complete non-metformin monotherapy (model 3). Table 3 shows the main results (Supplementary Material 2). In each analysis, the adjusted covariates were sex, age, income level, BMI, FBG, total cholesterol, hypertension, SGPT (ALT), CCI, smoking status, and follow-up period.

Model 1: The OR for any medication prescription (≥180 days) within LMT versus otherwise was 0.75 (95% CI, 0.67 to 0.84, p<0.001).

Model 2: Cancer incidence was significantly lower for metformin monotherapy, metformin combination therapy, and non-metformin monotherapy than for no prescription ever. The ORs were as follows: 0.66 (95% CI, 0.51 to 0.83; p<0.001), 0.75 (95% CI, 0.64 to 0.88; p<0.001), and 0.82 (95% CI, 0.68 to 0.99; p<0.05) for metformin monotherapy, metformin combination therapy, and non-metformin monotherapy, respectively. The pattern of late prescription with poor compliance showed significantly higher incidence at the significance level of 0.1, and the OR was 1.24 (95% CI, 0.97 to 1.58, p<0.1).

Model 3: The OR for complete metformin monotherapy against complete non-metformin monotherapy was 0.63 (95% CI, 0.41 to 0.99, p<0.05).

## DISCUSSION

We found that patients with early antidiabetic medication use (with compliance) had an approximately 25% lower risk of cancer than those who did not (OR, 0.75). These findings are consistent with previous studies showing that early treatment of newly diagnosed patients can help prevent diabetes complications [7–9].

In this study, more than 50% of patients were not prescribed antidiabetic medication for at least 2 years after their diabetes diagnosis. It can be assumed that many patients with diabetes patients take no action or only make lifestyle changes such as exercise and diet for a while after being diagnosed with diabetes. However, lifestyle changes in patients with diabetes have been reported to be negligible based on data from a large population-based sample [10]. Therefore, early medication treatment is necessary to prevent diabetes complications, including cancer.

For patients who received their first prescription late (more than 2 years after being diagnosed with diabetes), compliance with prescriptions was found to be important for cancer prevention. A noteworthy finding is that if a patient was given the first medication prescription late, it is highly likely that diabetes management using methods such as lifestyle modification had not been effective or the severity of diabetes had escalated during the first 2 years.

In this study, cancer incidence for the patients who received their first prescription late was significantly higher than that of non-users (the no prescription ever group) when patients did not comply with the prescriptions (OR, 1.24). These results not only support that poor adherence to prescribed drugs is an important medical problem in previous studies [11,12], but also indicate the importance of prescription compliance when the timing of prescriptions is delayed. Therefore, in addition to the aforementioned importance of early medication compliance, patients must comply with their prescribed medication when prescriptions are delayed.

Metformin, a drug used to treat type 2 diabetes, is the most widely prescribed diabetes medication worldwide and has many beneficial effects, including anticancer effects [14–16]. Numerous studies have claimed that metformin could suppress the growth of cancer cells in multiple types of cancer including gastroenterological, colorectal, liver, lung, breast, and pancreatic cancer [17–24,46–48].

However, some studies had conflicting results regarding the anticancer effects of metformin. No clear association was found between metformin use and all-sites cancer including colon, bladder, lung, and breast cancer, but potentially important confounders such as BMI were not considered in that study [28]. Metformin initiators did not have a reduced risk of breast cancer compared with a clinical alternative in older females, but that study had a short follow-up time [29]. Metformin use was not associated with lung cancer in patients with type 2 diabetes based on a conditional logistic regression model, but that study did not consider compliance with the prescribed treatment [30].

Most studies on the cancer-preventive effects of metformin have been based on observational studies with methodological biases, which has been pointed out as a problem. Observational studies need to be designed using rigorous methods to reduce the potential for bias [25,31]. LMT analysis is a method to control immortal time bias alongside time-dependent analyses [36,37]. The application of an LMT analysis with a suitable LMT is simpler than a time-dependent analysis, with easier interpretation of the results, and it has confirmed validity for controlling immortal time bias [43].

In this study, the incidence of cancer was significantly lower with metformin monotherapy and metformin combination therapy (OR, 0.66 and 0.75, respectively) compared to that of non-use (the no prescription ever group) based on the LMT method. As evidence to support the anticancer effect of metformin, the effect was approximately 34% and 25% in the metformin monotherapy and metformin combination groups, respectively. Thus, the anticancer effect of metformin was verified in this study using the LMT analysis to control for immortal time bias. Additionally, this study was a long-term cohort study that considered potentially important confounding factors such as BMI and prescription compliance.

Yoshida et al. [49] suggested using the active-comparator design, in which treatment groups with similar treatment indications are selected by comparing the drug of interest to another commonly used agent, rather than a non-user group. This is because non-users are not suitable as the control group in analyzing drug effects, as they are likely to vary in drug stage from one extreme to the other, with the severity of the disease ranging from mild to extremely severe.

We defined complete monotherapy as early administration of a single medication followed by no other drug administration than this medication thereafter. Therefore, patients with complete monotherapy are very likely to have a relatively low level of diabetes severity. Complete metformin monotherapy was associated with a significantly lower risk of cancer (OR, 0.63) compared to complete non-metformin monotherapy in this study, indicating an approximately 37% anticancer effect. In fact, the influence of sex and age was high, while the influence of CCI or follow-up period was relatively low and important confounders such as BMI, total cholesterol, and SGPT (ALT) did not show significant differences (Supplementary Material 2). This indicates that complete non-metformin monotherapy and complete metformin monotherapy are controlled at a similar (mild) condition of diabetes severity.

Furthermore, 31.8% of patients who started metformin monotherapy switched to another drug, and 62.0% of patients who started non-metformin monotherapy switched to another drug later in the present study. This aligns with the results of a previous study where metformin showed higher durability than non-metformin [50]. In other words, metformin may be used to ensure more stable and prolonged effects in diabetes treatment than other drugs.

The study has several limitations. First, a fixed LMT was used without considering the sensitivity of the LMT. If the LMT is of long duration, the statistical power of analysis may decrease because of the reduced number of samples due to death (or event occurrence) during this period [37]. In this study, patients with at least 2 years of follow-ups were targeted, as cancer incidence has a long latency period and diabetes is a chronic disease with relatively slow progression. Accordingly, the LMT was set to 2 years. The actual number of patients excluded due to the LMT setting was 2,040 based on the incidence of cancer within the LMT, but it was reasonable to exclude them because they had the potential for cancer-causing diabetes [40]. Furthermore, this study considered both pre- and post-LMT prescription status in order to compensate for the sensitivity of the LMT. Nonetheless, further studies should examine the sensitivity of LMT in greater detail.

Second, although several efforts, including LMT analysis, have controlled potential bias, residual confounding from other sources is still possible. For example, diabetes severity may vary between treatment groups. The metformin monotherapy group may have less severe disease than the non-metformin monotherapy group. In this study, we compared the complete metformin monotherapy group with the complete non-metformin monotherapy group, and the results confirmed the anticancer effect of metformin along with the similar (mild) severity between the 2 groups. However, a more detailed study considering various levels of diabetes severity is needed. Moreover, medication exposure was defined only considering total prescription days of the medication, and doses of drugs were not considered. Furthermore, the patients prescribed first after the LMT may have developed cancer before taking the medication. In future studies, an analysis should be conducted after removing these patients as well.

Third, it was assumed that diabetes was not completely curable in this study. That is, patients were considered to have diabetes during the entire follow-up period. In particular, some non-users may have had a lower risk of cancer compared to some other treatment groups because diabetes was cured or so mild that medication was not required.

Fourth, the cancer incidence rate may have been overestimated in this study because cancer incidence was defined only by the cancer diagnosis code.

In conclusion, the prescription medication with compliance within 2 years of the first diabetes diagnosis led to a cancer prevention effect of approximately 25%. The risk of cancer increased if patients did not comply with medication when prescription was delayed. Therefore, prescribing medication early is important, and immediate compliance with medication, regardless of the time of prescription, is also important. The cancer prevention effect of metformin use (approximately 25–34%) was verified through an LMT analysis that controlled immortal time bias. Furthermore, when starting with an initial single drug and continuing the drug without change through the entire follow-up period, metformin showed more durability and higher efficacy (approximately 37%) at preventing cancer than non-metformin drugs.

It is important for doctors to prescribe antidiabetic medication early. Management and education are necessary to ensure that patients with diabetes comply with their prescribed medications regardless of prescription timing. Metformin monotherapy or metformin combination therapy is recommended as an early treatment.

## Figures and Tables

**Table 1:** Frequency of sample characteristics

Characteristics	n (%)
Sex	
Male	18,980 (52.6)
Female	17,119 (47.4)

Age (yr)	
30–49	10,673 (29.6)
50–69	20,482 (56.7)
70–89	4,944 (13.7)

Income level	
Low	11,334 (31.4)
Mid	13,940 (38.6)
High	10,825 (30.0)

BMI (kg/m^2^)	
Normal (<25)	9,628 (26.7)
Overweight (25–30)	9,455 (26.2)
Obesity (≥30)	17,016 (47.1)

FBG (mg/dL)	
Normal	26,419 (73.2)
High (>126)	9,680 (26.8)

Total cholesterol (mg/dL)	
Normal	31,888 (88.3)
High (≥240)	4,211 (11.7)

Hypertension	
No	16,315 (45.2)
Yes	19,784 (54.8)

SGPT (ALT) (U/L)	
Normal	30,319 (84.0)
High (≥40)	5,780 (16.0)

CCI	
0	12,007 (33.3)
1	11,236 (31.1)
2	4,559 (12.6)
≥3	8,297 (23.0)

Smoking status	
Never	22,363 (61.9)
Former	6,152 (17.0)
Current	7,584 (21.0)

Follow-up period (yr)	
<5	12,207 (33.8)
5–7	8,656 (24.0)
7–9	10,152 (28.1)
≥9	5,084 (14.1)

Cancer	
No	34,216 (94.8)
Yes	1,883 (5.2)

BMI, body mass index; FBG, fasting blood glucose; SGPT (ALT), serum glutamic pyruvic transaminase (alanine aminotransferase); CCI, Charlson comorbidity index.

**Table 2:** Medication prescription status

Medication status	Before the LMT^1^	After the LMT^1^	Total	Cancer
Insulin	Insulin administration regardless of any medication use	-	434 (1.2)	32 (7.4)

Metformin monotherapy				
Complete	Metformin only	Metformin only or all <180 d	1,729 (4.8)	41 (2.4)
Incomplete	Metformin only	Non-metformin	807 (2.2)	39 (4.8)

Non-metformin monotherapy				
Complete	Non-metformin only	The same drug only or all <180 d	1,160 (3.2)	57 (4.9)
Incomplete	Non-metformin only	Another medication	1,893 (5.2)	108 (5.7)

Metformin combination	Metformin & non-metformin	No restriction	5,836 (16.2)	230 (3.9)

Non-metformin combination	At least 2 non-metformin	No restriction	473 (1.3)	27 (5.7)

Early prescription and poor compliance	Prescription but all <180 d	No restriction	4,423 (12.2)	242 (5.5)

Late prescription with compliance	Non-prescription	Prescription with ≥180 d	3,161 (8.8)	222 (7.0)

Late prescription with poor compliance	Non-prescription	Prescription but all <180 d	1,082 (3.0)	80 (7.4)

No prescription ever	Non-prescription	Non-prescription	15,101 (41.8)	805 (5.3)

Total			36,099 (100)	1,883 (5.2)

Values are presented as number (%).

LMT, landmark time.

1 2 Years after first diabetes diagnosis.

**Table 3:** Odds ratios^1^ of cancer incidence according to prescription medication status

Medication status	Model 1	Model 2	Model 3
Early prescription with compliance	0.75 (0.76, 0.84)^***^	-	-
Metformin monotherapy	-	0.66 (0.51, 0.83)^**^	-
Metformin combination	-	0.75 (0.64, 0.88)^***^	-
Non-metformin monotherapy	-	0.82 (0.68, 0.99)^*^	-
Non-metformin combination	-	0.90 (0.58, 1.32)	-

No early prescription with compliance	1.00 (reference)	-	-
Early prescription with poor compliance	-	1.01 (0.87, 1.18)	-
Late prescription with compliance	-	1.02 (0.87, 1.20)	-
Late prescription with poor compliance	-	1.24 (0.97, 1.58)^+^	-
No prescription ever	-	1.00 (reference)	-

Complete metformin monotherapy	-	-	0.63 (0.41, 0.99)^*^

Complete non-metformin monotherapy	-	-	1.00 (reference)

Values are presented as odds ratio (95% confidence interval).

1 Adjusted for all variables in Table 1 except the target variable (cancer).

+ p<0.1,

* p<0.05,

** p<0.01

*** p<0.001.
