# Supplementary material for: Effects of early medication treatment and metformin use for cancer prevention in diabetes patients: a nationwide sample cohort study in Korea using extended landmark time analysis
Source: Epidemiol Health. 2021 Dec 17;43:e2021103. doi: 10.4178/epih.e2021103 (PMC8920735; doi:10.4178/epih.e2021103)
Supplement: Supplementary file 1 [file epih-43-e2021103-suppl.docx]

**Supplementary Material 1. Medication list with generic codes**

| Generic code | ATC^1^ code | Ingredient | name |
| --- | --- | --- | --- |
| 100601ATB | A10BF | acarbose | Alpha-glucosidase inhibitors |
| 165402ATB | A10BB | glibenclamide | Sulfonylureas |
| 165602ATB | A10BB | gliclazide | Sulfonylureas |
| 165701ATB | A10BB | glimepiride | Sulfonylureas |
| 165801ATB | A10BB | glipizide | Sulfonylureas |
| 170130BIJ | A10AC | insulin (human) | Insulin |
| 170430BIJ | A10AD | insulin (human) | Insulin |
| 175330BIJ | A10AB | insulin_lispro | Insulin |
| 191501ATB | A10BA | metformin | Metformin |
| 249001ATB | A10BF | voglibose | Alpha-glucosidase inhibitors |
| 379501ATB | A10BX | repaglinide | Meglitinides |
| 406201ATB | A10BF | miglitol | Alpha-glucosidase inhibitors |
| 430201ATB | A10BX | nateglinide | Meglitinides |
| 431901ATB | A10BG | pioglitazone | TZD |
| 441330BIJ | A10AB | insulin_aspart | Insulin |
| 443400ATB | A10BD | metformin&sulfonylureas | Metformin&Sulfonylureas |
| 443500ATB | A10BD | metformin&sulfonylureas | Metformin&Sulfonylureas |
| 461830BIJ | A10AE | insulin_glargine | Insulin |
| 471900ATB | A10BD | metformin&sulfonylureas | Metformin&Sulfonylureas |
| 474200ATB | A10BD | sulfonylureas&metformin | Metformin&Sulfonylureas |
| 474300ATB | A10BD | sulfonylureas&metformin | Metformin&Sulfonylureas |
| 484930BIJ | A10AB | insulin_glulisine | Insulin |
| 486101ATB | A10BX | mitiglinide | Meglitinides |
| 488730BIJ | A10AE | insulin_detemir | Insulin |
| 497200ATB | A10BD | metformin&sulfonylureas | Metformin&Sulfonylureas |
| 498100ATB | A10BD | pioglitazone&metformin | Metformin&TZD |
| 498600ATB | A10BD | sulfonylureas&metformin | Metformin&Sulfonylureas |
| 500801ATB | A10BH | vildagliptin | DPP4i |
| 501101ATB | A10BH | sitagliptin | DPP4i |
| 502300ATB | A10BD | sitagliptin&metformin | Metformin&DPP4i |
| 502900ATB | A10BD | sitagliptin&metformin | Metformin&DPP4i |
| 507000ATB | A10BD | vildagliptin&metformin | Metformin&DPP4i |
| 507100ATB | A10BD | vildagliptin&metformin | Metformin&DPP4i |
| 512130BIJ | A10BX | exenatide | Meglitinides |
| 513700ATB | A10BD | sitagliptin&metformin | Metformin&DPP4i |
| 518500ATR | A10BD | saxagliptin&metformin | Metformin&DPP4i |
| 518600ATR | A10BD | saxagliptin&metformin | Metformin&DPP4i |
| 518800ATB | A10BD | combinations | Metformin&Alpha-glucosidase inhibitors |
| 519600ATB | A10BD | vildagliptin&metformin | Metformin&DPP4i |
| 520500ATB | A10BD | linagliptin&metformin | Metformin&DPP4i |
| 520600ATB | A10BD | linagliptin&metformin | Metformin&DPP4i |
| 520700ATB | A10BD | linagliptin&metformin | Metformin&DPP4i |
| 523600ATB | A10BD | combinations | Metformin&Alpha-glucosidase inhibitors |
| 523700ATB | A10BD | combinations | Metformin&Alpha-glucosidase inhibitors |
| 523800ATR | A10BD | gemigliptin&metformin | Metformin&DPP4i |
| 524700ATR | A10BD | sitagliptin&metformin | Metformin&DPP4i |
| 525500ATB | A10BD | pioglitazone&glimepiride | Sulfonylureas&TZD |
| 525600ATB | A10BD | pioglitazone&glimepiride | Sulfonylureas&TZD |
| 525901ATB | A10BG | Thiazolidinediones | TZD |
| 527301ATB | A10BK | dapagliflozin | SGLT2 inhibitors |
| 613301ATB | A10BH | saxagliptin | DPP4i |
| 616401ATB | A10BH | linagliptin | DPP4i |
| 619101ATB | A10BH | gemigliptin | Sulfonylureas |
| 624201ATB | A10BH | alogliptin | DPP4i |
| 626630BIJ | A10BX | lixisenatide | Meglitinides |
| 626700BIJ | A10AD | insulin_degludec&insulin_aspart | Insulin |
| 626830BIJ | A10AE | insulin_degludec | Insulin |
| 627301ATB | A10BH | dpp4 | DPP4i |
| 628201ATB | A10BK | empagliflozin | SGLT2 inhibitors |
| 630300ATB | A10BD | alogliptin&pioglitazone | DPP4i&TZD |
| 630400ATB | A10BD | alogliptin&pioglitazone | DPP4i&TZD |
| 630500ATB | A10BD | alogliptin&pioglitazone | DPP4i&TZD |
| 630600ATB | A10BD | alogliptin&pioglitazone | DPP4i&TZD |
| 631900ATB | A10BD | combinations | Metformin&Meglitinides |
| 632000ATR | A10BD | gemigliptin&metformin | Metformin&Sulfonylureas |
| 632100ATB | A10BD | repaglinide&metformin | Metformin&Meglitinides |
| 635600ATB | A10BD | alogliptin&metformin | Metformin&DPP4i |
| 635700ATB | A10BD | alogliptin&metformin | Metformin&DPP4i |
| 636101ATB | A10BK | other | SGLT2 inhibitors |
| 637200ATB | A10BD | repaglinide&metformin | Metformin&Meglitinides |
| 639601ATB | A10BH | dpp4 | DPP4i |
| 639701BIJ | A10BX | dulaglutide | Meglitinides |
| 639800ATR | A10BD | dapagliflozin&metformin | Metformin&SGLT2 inhibitors |
| 641400ATR | A10BD | dapagliflozin&metformin | Metformin&SGLT2 inhibitors |
| 641800ATR | A10BH | dpp4 | DPP4i |
| 641900ATR | A10BH | dpp4 | DPP4i |
| 642000ATR | A10BH | dpp4 | DPP4i |
| 644501BIJ | A10BJ | albiglutide | GLP-1 |
| 644900ATB | A10BX | nateglinide&metformin | Metformin&Meglitinides |
| 645000ATR | A10BD | gemigliptin&metformin | Metformin&Sulfonylureas |
| 645301ATB | A10BH | dpp4 | DPP4i |
| 648400ATB | A10BH | dpp4 | DPP4i |
| 648500ATB | A10BH | dpp4 | DPP4i |
| 648600ATB | A10BH | dpp4 | DPP4i |
| 649000ATB | A10BD | metformin&empagliflozin | Metformin&SGLT2 inhibitors |
| 649100ATB | A10BD | metformin&empagliflozin | Metformin&SGLT2 inhibitors |
| 649200ATB | A10BD | metformin&empagliflozin | Metformin&SGLT2 inhibitors |
| 649300ATB | A10BD | metformin&empagliflozin | Metformin&SGLT2 inhibitors |
| 649400ATB | A10BD | metformin&empagliflozin | Metformin&SGLT2 inhibitors |
| 649500ATB | A10BD | metformin&empagliflozin | Metformin&SGLT2 inhibitors |
| 649900ATR | A10BH | dpp4 | DPP4i |
| 650000ATR | A10BH | dpp4 | DPP4i |
| 650100ATR | A10BH | dpp4 | DPP4i |
| 653800ATR | A10BD | combinations | Metformin&TZD |
| 653900ATR | A10BD | combinations | Metformin&TZD |
| 654000ATR | A10BD | combinations | Metformin&TZD |
| 654100ATR | A10BD | gemigliptin&metformin | Metformin&Sulfonylureas |
| 655700ATR | A10BD | combinations | Metformin&TZD |
| 664600ATB | A10BH | gemigliptin&rosuvastatin | Sulfonylureas&DPP4i |
| 664700ATB | A10BH | gemigliptin&rosuvastatin | Sulfonylureas&DPP4i |
| 664800ATB | A10BH | gemigliptin&rosuvastatin | Sulfonylureas&DPP4i |

^1^Anatomical therapeutic chemical classification system

**Supplementary Material 2.** Results of logistic regression analyses for *Model 1*, *Mode1 2*, *and Model 3*.

| Variables | Model 1  OR CI | | Model 2  OR CI | | Model 3  OR CI | |
| --- | --- | --- | --- | --- | --- | --- |
| Sex (female) | **0.57***** | 0.50-0.64 | **0.57***** | 0.51-0.64 | **0.41**** | 0.24-0.70 |
| Age (50-69) | **2.18***** | 1.91-2.51 | **2.18***** | 1.90-2.51 | **4.14***** | 1.98-10.12 |
| Age (70-89) | **2.78***** | 2.33-3.31 | **2.77***** | 2.32-3.30 | **4.32**** | 1.78-11.68 |
| Income level (mid) | 1.01 | 0.90-1.14 | 1.02 | 0.91-1.14 | 0.77 | 0.48-1.25 |
| Income level (high) | 1.02 | 0.90-1.15 | 1.02 | 0.90-1.15 | 0.78 | 0.46-1.31 |
| BMI (overweight) | 0.91 | 0.80-1.03 | 0.90 | 0.79-1.03 | 0.65 | 0.37-1.14 |
| BMI (obesity) | **0.90^+^** | 0.80-1.02 | **0.90^+^** | 0.80-1.02 | 0.69 | 0.42-1.13 |
| FBG (high) | 0.98 | 0.87-1.11 | 0.97 | 0.86-1.10 | 0.80 | 0.49-1.28 |
| Total cholesterol (high) | **0.87^+^** | 0.73-1.02 | **0.86^+^** | 0.73-1.01 | 1.02 | 0.47-1.99 |
| Hypertension (yes) | 0.9 | 0.86-1.06 | 0.95 | 0.86-1.06 | 1.21 | 0.77-1.93 |
| SGPT(ALT) (high) | **1.16*** | 1.01-1.32 | **1.15*** | 1.00-1.32 | 1.28 | 0.71-2.19 |
| CCI (1) | **1.70***** | 1.48-1.96 | **1.70***** | 1.48-1.95 | 1.44 | 0.83-2.50 |
| CCI (2) | **1.97***** | 1.67-2.33 | **1.97***** | 1.66-2.32 | 0.87 | 0.36-1.89 |
| CCI (≥3) | **2.45***** | 2.13-2.82 | **2.43***** | 2.11-2.81 | **1.75*** | 1.02-3.06 |
| Smoking status (former) | 0.98 | 0.85-1.14 | 0.99 | 0.85-1.14 | 0.83 | 0.45-1.52 |
| Smoking status (current) | 0.93 | 0.80-1.07 | 0.93 | 0.80-1.07 | 1.09 | 0.61-1.93 |
| Follow-up period (5-7) | **1.64***** | 1.41-1.89 | **1.62***** | 1.53-2.05 | **1.61^+^** | 0.93-2.72 |
| Follow-up period (7-9) | **2.40***** | 2.10-2.75 | **2.36***** | 2.30-3.05 | **2.10*** | 1.21-3.60 |
| Follow-up period (≥9) | **2.52***** | 2.16-2.94 | **2.47***** | 2.66-3.52 | 1.61 | 0.67-3.48 |
| Metformin monotherapy | **0.75***** | 0.67-0.84 | **0.66***** | 0.51-0.83 | x | x |
| Metformin combination |  |  | **0.75***** | 0.64-0.88 | x | x |
| Non-metformin monotherapy |  |  | **0.82*** | 0.68-0.99 | x | x |
| Non-metformin combination |  |  | 0.90 | 0.58-1.32 | x | x |
| Early prescription with poor compliance | 1.00  [Reference] | | 1.01 | 0.86-1.18 | x | x |
| Late prescription with compliance |  |  | 1.02 | 0.87-1.20 | x | x |
| Late prescription with poor compliance |  |  | **1.24^+^** | 0.97-1.58 | x | x |
| No prescription ever |  |  | 1.00 [Reference] | | x | x |
| Complete metformin monotherapy | x | x | x | x | **0.63*** | 0.41-0.99 |
| Complete non-metformin monotherapy | x | x | x | x | 1.00 [Reference] | |

**^+^** p<0.1, *p<0.05, **p<0.01, ***p<0.001. OR, adjusted odds ratio. CI, 95% confidence interval.

BMI, Body Mass Index; CCI, Charlson Comobidity Index; FBG, Fasting Blood Glucose; SGPT(ALT), Serum Glutamic Pyruvic Transaminase (Alanine Aminotransferase).
